# ﻿Two new rove beetle genera in Staphylininae that reduce “*Heterothops*” and “*Quedius*” taxonomic wastebaskets (Coleoptera, Staphylinidae)

**DOI:** 10.3897/zookeys.1218.135558

**Published:** 2024-11-15

**Authors:** José L. Reyes-Hernández, Alexey Solodovnikov

**Affiliations:** 1 Natural History Museum of Denmark, University of Copenhagen, Zoological Museum, Universitetsparken 15, 2100, Copenhagen, Denmark University of Copenhagen Copenhagen Denmark

**Keywords:** *Cheilocolpus*, *Chiquiticus* gen. nov., *Cyrtoquedius*, Neotropical fauna, new combinations, new lineages, *Nitidocolpus* gen. nov., taxonomy

## Abstract

The here-provided description of the new genera *Chiquiticus***gen. nov.** and *Nitidocolpus***gen. nov.** was necessitated by a phylogenetic study of Staphylininae (to be published separately), which will be used for the proper characterization of their respective new suprageneric lineages in an upcoming update of the higher classification of this subfamily. Both new genera are erected for species that had been previously described but misplaced in the highly polyphyletic “taxonomic wastebasket” genera *Heterothops* (Amblyopinina) and *Quedius* (Quediina), resulting in the following new combinations: *Chiquiticusarizonicus* (Smetana, 1971), **comb. nov.** ex. *Heterothops*; *Chiquiticuscampbelli* (Smetana, 1971), **comb. nov.** ex. *Heterothops*; †*Chiquiticuscornelli* (Chatzimanolis & Engel, 2013), **comb. nov.** ex. *Heterothops*; *Chiquiticusgemellus* (Smetana, 1971), **comb. nov.** ex. *Heterothops*; †*Chiquiticusinfernalis* (Chatzimanolis & Engel, 2013), **comb. nov.** ex. *Heterothops*; *Chiquiticusoccidentis* (Casey, 1886), **comb. nov.** ex. *Heterothops*; *Chiquiticuspusio* (LeConte, J. L., 1863), **comb. nov.** ex. *Heterothops*; *Chiquiticusrambouseki* (Blackwelder, 1943), **comb. nov.** ex. *Heterothops*; *Nitidocolpusaurofasciatus* (Bernhauer, 1917), **comb. nov.** ex. *Quedius*; *Nitidocolpuschampioni* (Sharp, 1884), **comb. nov.** ex. *Quedius*; *Nitidocolpuscolumbinus* (Bernhauer, 1917), **comb. nov.** ex. *Quedius*; *Nitidocolpusgermaini* (Bernhauer, 1917), **comb. nov.** ex. *Quedius*; *Nitidocolpusillatus* (Sharp, 1884), **comb. nov.** ex. *Quedius*; *Nitidocolpuslaeticulus* (Sharp, 1884), **comb. nov.** ex. *Quedius*; *Nitidocolpustriangulum* (Fauvel, 1891), **comb. nov.** ex. *Quedius*. Additionally, several more Neotropical *Quedius* species, which resemble *Nitidocolpus*, have been revised and transferred to the amblyopinine genus *Cheilocolpus* or the cyrtoquediine genus *Cyrtoquedius*, with the following new combinations: *Cheilocolpusforsteri* (Scheerpeltz, 1960), **comb. nov.** ex. *Quedius*; *Cheilocolpusspeciosus* (Bernhauer, 1917), **comb. nov.** ex. *Quedius*; *Cheilocolpusviridulus* (Erichson, 1840), **comb. nov.** ex. *Quedius*; *Cyrtoquediusviridipennis* (Fauvel, 1891), **comb. nov.** ex. *Quedius*. The undescribed species diversity of the newly described genera is also highlighted.

## ﻿Introduction

**Figure 1.**
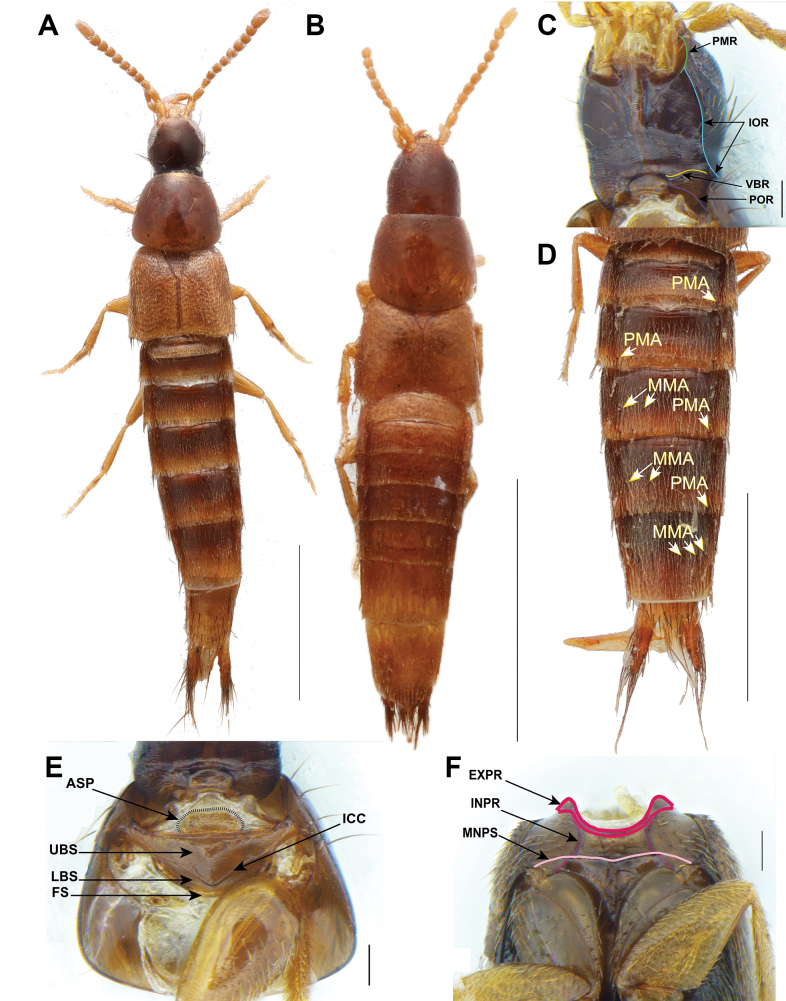
Some *Chiquiticus* species and characters **A**C.sp.nr.pusio**B***C.rambouseki* (Blackwelder, 1943) **C***C.pusio* (LeConte, J. L., 1863), head ventral view **D***C.pusio*, abdomen **E***C.pusio*, prosternum **F***C.pusio*, mesothorax. Abbreviations: ASP, antesternal plate; EXPR, external part of prepectus; FS, profurcasternum; ICC, intercoxal carina of the probasisternum; INPR, internal part of prepectus; IOR, infraorbital ridge; LBS, lower probasisternum; MMA, medial macroseta; MNPS, mesanapleural sutures; PMA, posteromarginal large macrosetae; PMR, postmandibular ridge; POR, postoccipital ridge; UBS, upper probasisternum; VBR, ventral basal ridge. Scale bars: 1 mm (**A, B, D**); 0.1 mm (**C, E, F**).

**Figure 2.**
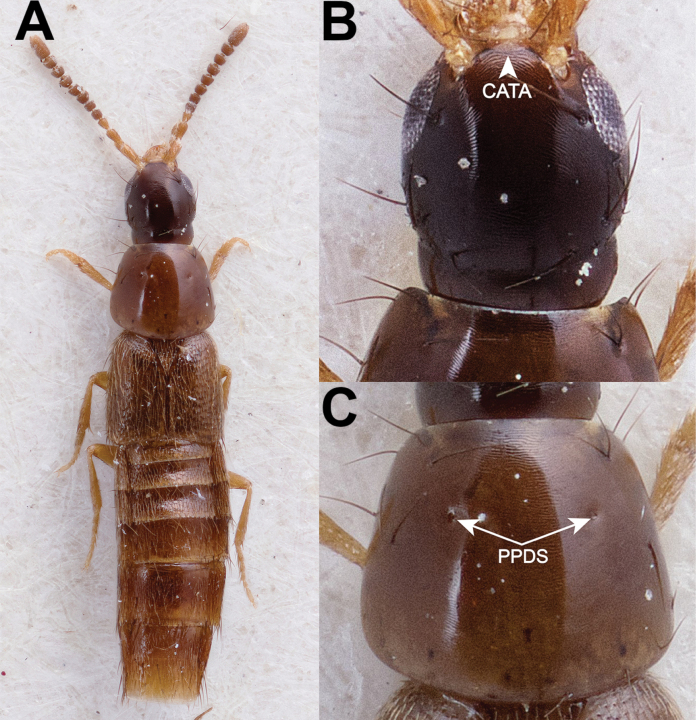
*Chiquiticusarizonicus* (Smetana, 1971) (Holotype #CNC935711) **A** dorsal habitus **B** head **C** pronotum. Abbreviations: CATA, connection of the anterior tentorial arms (frontoclypeal “suture”); PPDS, paired punctures on dorsal series. Photos of # CNC935711 (J. Buffam, Canadian National Collection of Insects, Arachnids and Nematodes).

**Figure 3.**
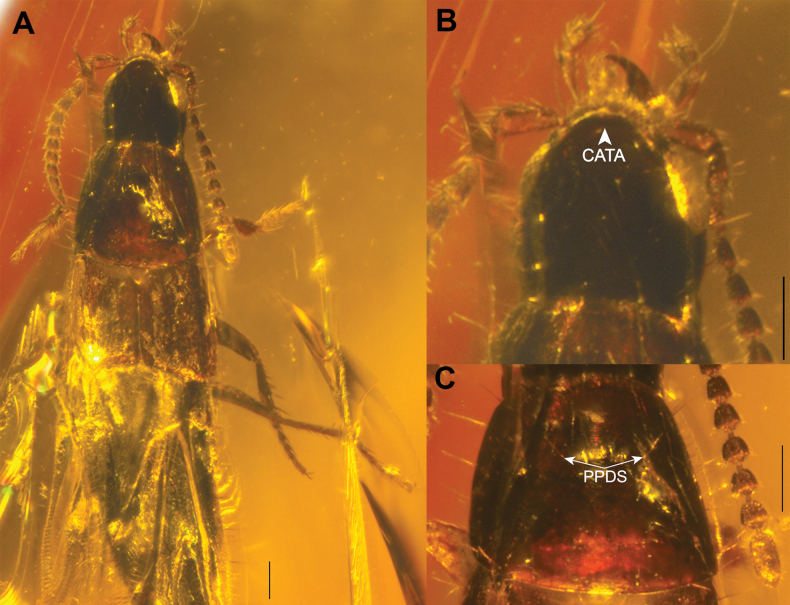
†*Chiquiticuscornelli* (Chatzimanolis & Engel, 2013) (paratype AMNH DR-10-1214) **A** dorsal habitus **B** head **C** pronotum. Abbreviations: CATA, connection of the anterior tentorial arms (frontoclypeal “suture”); PPDS, paired punctures on dorsal series. Photo of AMNH DR-10-1214 (A. Pierwola, American Museum of Natural History). Scale bars: 0.1 mm.

**Figure 4.**
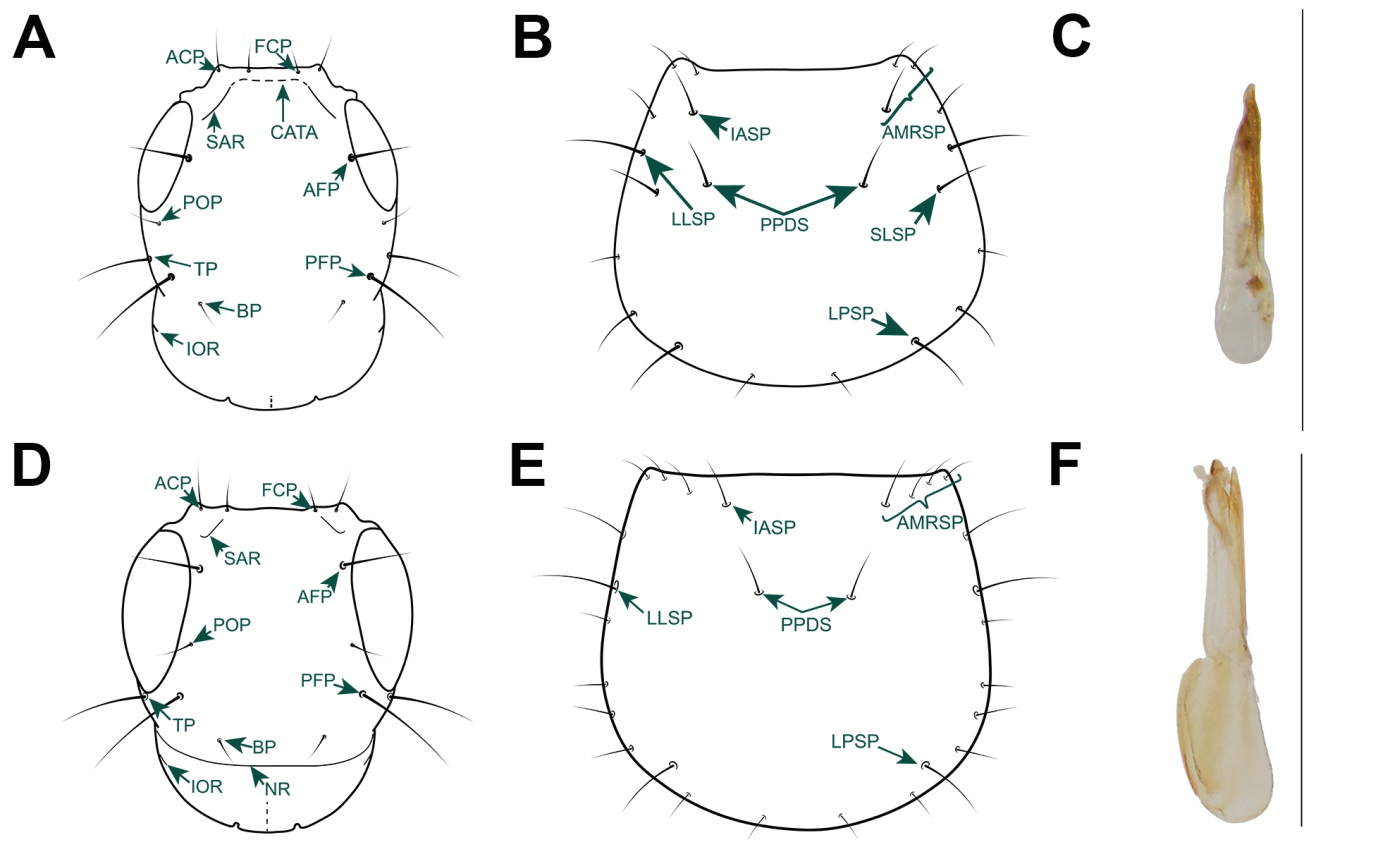
Morphological details of *Chiquiticus* and *Heterothops***A–C**Chiquiticussp. aff.pusio from Panama **D–F***Heterothops* sp. undescribed from Costa Rica **A, D** head **B, E** pronotum **C, F** aedeagus in lateral view, the scale to the right is equal to 1 mm. Abbreviations: ACP, anterolateral clypeal puncture; AFP, anterior frontal; AMRS, anterior marginal row of setiferous punctures; BP, basal puncture; CATA, connection of the anterior tentorial arms (frontoclypeal “suture”); FCP, frontoclypeal puncture; IASP, internalmost setiferous puncture of the anterior margin; IOR, infraorbital ridge; LLSP, large lateral setiferous puncture; LPSP, large posterior setiferous puncture; NR, nuchal ridge; PFP, posterior frontal puncture; POP, parocular punctures; PPDS, paired punctures on dorsal series; SAR, supra-antennal ridge; SLSP, sublateral setiferous puncture; TP, temporal puncture.

**Figure 5.**
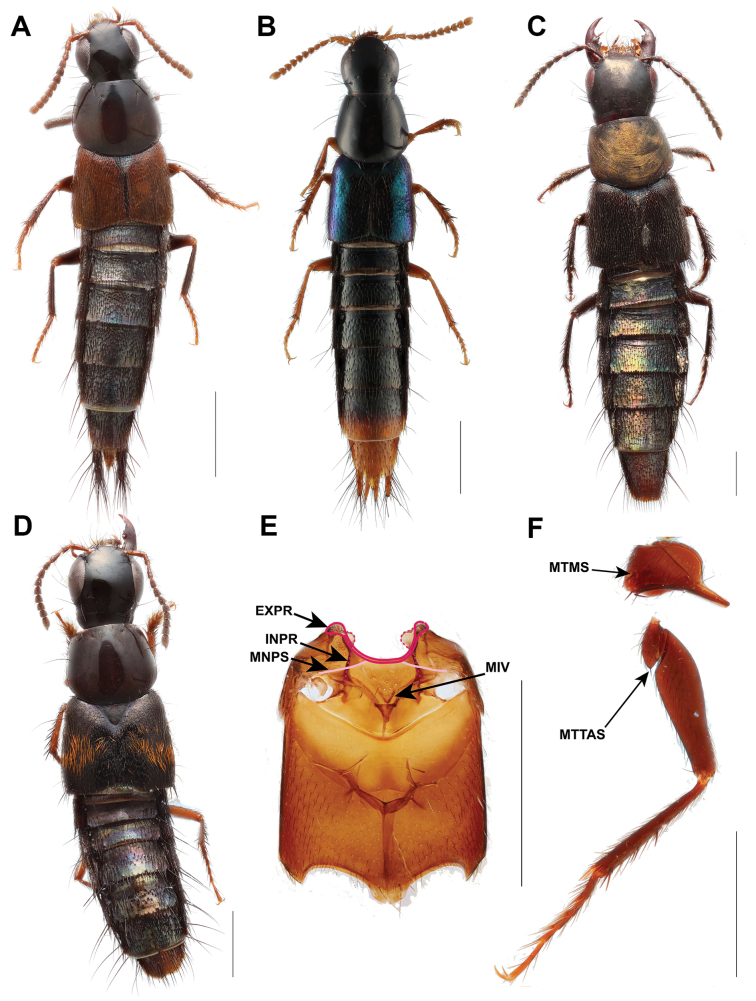
Some *Nitidocolpus* species and characters **A***N.illatus* (Sharp, 1884) **B***N.laeticulus* (Sharp, 1884) **C***N.germaini* (Bernhauer, 1917) **D***N.aurofasciatus* (Bernhauer, 1917) **E***N.illatus*, pterothoraces **F***N.columbinus* (Bernhauer, 1917), metacoxa and metatrochanter, dissected right leg in dorsal view (side faced to the abdomen). Abbreviations: EXPR, external part of prepectus; INPR, internal part of prepectus; MIV, mesobasisternal intercoxal process with V-shaped projection medially; MNPS, mesanapleural sutures; MTMS, metacoxa with dorsomedial spine; MTTAS, metatrochanter with dorsoapical spine. Photo B by J. Jenkins Shaw. Scale bars: 1 mm.

**Figure 6.**
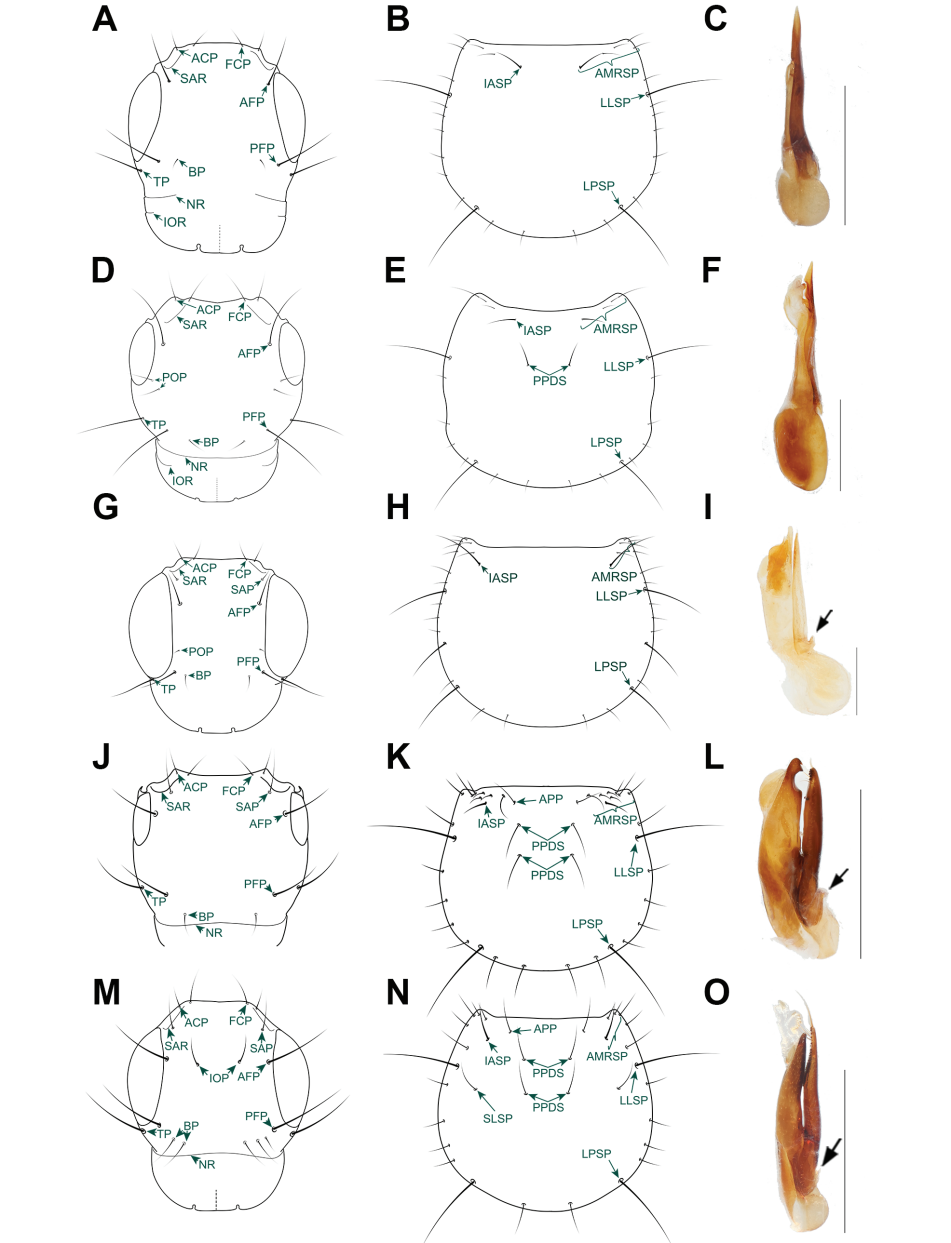
Morphological details of *Nitidocolpus* and other relevant genera of Staphylininae**A–C***Nitidocolpuscolumbinus***D–F***Cheilocolpusviridulus***G–I***Cyrtoquedius* sp. **J–L**Quedionuchusnr.impunctus**M–O**Quediusnr.advena. **A, D, G, J, M** head **B, E, H, K, N** pronotum **C, F, I, L, O** aedeagus in lateral view, the black arrow pointing down indicates the basal projection of the paramere. Abbreviations: ACP, anterolateral clypeal puncture; AFP, anterior frontal; AMRSP, anterior marginal row of setiferous punctures; APP, additional paired punctures adjacent to AMRSP; BP, basal puncture; FCP, frontoclypeal puncture; IASP, internalmost setiferous puncture of the anterior margin; IOP, interocular punctures; IOR, infraorbital ridge; LLSP, large lateral setiferous puncture; LPSP, large posterior setiferous puncture; NR, nuchal ridge; PFP, posterior frontal puncture; POP, parocular punctures; PPDS, paired punctures on dorsal series; SAP, supra-antennal puncture; SAR, supra-antennal ridge; SLSP, sublateral setiferous puncture; TP, temporal puncture. Scale bars: 1 mm.

During a large-scale systematic study of Staphylininae rove beetles (in the broad sense of Newton 2022), which integrates phylogenomic, morphological, and biogeographic evidence for the first time (Reyes-Hernández et al. in prep.), we discovered two new genera, *Chiquiticus* gen. nov. (Figs 1–3, 4A–C) and *Nitidocolpus* gen. nov. (Figs 5, 6A–C), both from the New World. These genera represent two previously unrecognized phylogenetic lineages within Staphylininae. The formal recognition of these lineages addresses a taxonomic gap, with further details to be presented in a forthcoming paper by Reyes-Hernández et al. (in prep.). Currently, both new genera are classified as Staphylininae*incertae sedis*.

*Chiquiticus* gen. nov. belongs to a newly recovered lineage that also includes the genus *Ctenandropus* Cameron, 1926 (now in the subtribe Amblyopinina of Staphylinini), the genus *Amazonothops* Jenkins Shaw, Orlov & Solodovnikov, 2020 (now Staphylinini*incertae sedis*), and a few extant and extinct (from Dominican amber) Nearctic and Neotropical species of the genus *Heterothops* Stephens, 1829 (now in the subtribe Amblyopinina). The small Australo-Asian genus *Ctenandropus* is morphologically and biogeographically peculiar and poorly known. *Amazonothops* is also a small Neotropical genus that was discovered only recently (Jenkins Shaw et al. 2020). *Chiquiticus* gen. nov. is erected for the few species of *Heterothops* that in fact do not belong to that genus and even to the subtribe Amblyopinina.

*Nitidocolpus* gen. nov. forms a monogeneric lineage comprising several Neotropical species of “*Quedius*” Stephens, 1829, primarily those related to *Q.columbinus* Bernhauer, 1917. The phylogenetic analysis of Reyes-Hernández et al. (in prep.) reveals that this lineage is distinct from true Quediina, as defined by Brunke et al. (2021). However, some species currently classified as *Quedius*, although superficially similar to *Q.columbinus* and its allies, were found to be more closely related to the genus *Cheilocolpus* Solier, 1849, both phylogenetically and morphologically. As a result, these species are transferred here to *Cheilocolpus*. Below, we present, justify, and discuss all these taxonomic novelties.

## ﻿Material and methods

The studied specimens are deposited in the following collections: **AMNH** (American Museum of Natural History, New York, USA; D.A. Grimaldi and A. Pierwola), **CNC** (Canadian National Collection, Ontario, Canada; A. Brunke), **FMNH** (Field Museum of Natural History, Chicago, Illinois, USA; M. Turcatel, A.F. Newton, M.K. Thayer), **IRSNB** (Institut Royal des Sciences Naturelles de Belgique, Brussels, Belgium; W. Dekoninck), **NHM** (The Natural History Museum, London, United Kingdom; M. Barclay and D. Telnov), **MCZ** (Museum of Comparative Zoology, Harvard University, Cambridge, Massachusetts, USA; C. Maier), and **MFNB** (Museum für Naturkunde an der Humboldt Universität, Berlin, Germany; B. Jäger).

Specimens were examined with a Leica M125 dissecting microscope (Leica Microsystems, Switzerland). Photographs were captured using a Canon 5D Mark III camera with a Canon MP-E 65mm f/2.8 1–5× macro lens (Canon, Japan) and a StackShot 3x (Cognisys, USA). Images were then stacked using Zerene Stacker (Zerene Systems, USA) with the PMax function. Further image processing, including cropping, lightening, drawing lines, and adding scales, was performed in Adobe Photoshop 2023.

Morphological terminology mainly follows Li and Zhou (2011), Brunke et al. (2019), and Reyes-Hernández et al. (2024), with the following modifications in prothorax and pterothorax terminology to align with that of Herman (2023). In the ventral view of the prothorax, what was formerly referred to as the basisternum and furcasternum, separated by the sternacostal ridge, is now termed the upper probasisternum (UBS, Fig. 1E) and lower probasisternum (LBS, Fig. 1E), respectively, separated by the intercoxal carina (ICC, Fig. 1E). In Staphylininae, the profurcasternum is a significantly reduced sclerite defined by an internal ridge connecting the apophyseal invaginations (Herman 2023). In the mesothorax (ventral view), the anterior margin is referred to as the external part of prepectus (EXPP, Fig. 1F), the sternopleural (anapleural) suture is termed as mesanapleural sutures (MNPS, Fig. 1F), and in the metathorax, the marginal carina of the mesocoxal acetabuli is called the pericoxal ridge. Regarding the frontoclypeal (epistomal) “suture”, here we clarify that it represents the connection of the anterior tentorial arms (CATA, Fig. 4A) inside the head capsule, which is superficially visible externally as a dark line (Figs 2B, 3B; see also fig. 7D, E in Reyes-Hernández et al. 2024). This “suture” or ridge is more clearly visible in teneral or chemically clarified specimens, while in highly sclerotized species it appears as a shallow line on the surface as well. The supra-antennal ridge (SAR) is the term used here for a structure referred to as the supra-antennal carina in the figure legend of Brunke (2022: fig. 3A, B) but described as the suprantennal ridge in the main text. It represents the fold of the anterior or anterolateral margin of the supra-antennal tubercle which may or may not be carinated (Figs 4, 6). Additionally, new characters are introduced and illustrated here, such as the anterolateral clypeal punctures (ACP, Figs 4, 6), which are setiferous punctures laterally adjacent to the frontoclypeal punctures on the dorsal portion of the head. On the abdominal segments, the medial macroseta (MMA, Fig. 1D) and posteromarginal large macroseta (PMA, Fig. 1D) are also indicated. In certain Staphylininae species, the base of the paramere (on its outer side around the area of its attachment to the median lobe) exhibits an upward or forward projection that is most distinctly visible in lateral view (Fig. 6I, L, O).

Abbreviations for measurements are as follows: EYL (eye length in lateral view), HL (head length from the apex of the clypeus to the nuchal ridge, or when the latter is dorsally absent, then to a hypothetical line joining the sides of the nuchal ridge or the groove marking the nuchal constriction), HW (head width at the widest point, including eyes), GL (gena length), NW (neck width at the widest point), PL (pronotum length along the median line), PW (pronotum width at the widest point), and TL (total length from the anterior margin of the clypeus to the posterior margin of segment VIII). All measurements were taken in millimeters using an ocular micrometer on a dissecting microscope.

## ﻿Results

### ﻿Family Staphylinidae Latreille, 1802

**Subfamily Staphylininae Latreille, 1802**

**Tribe incertae sedis**

#### ﻿Genus Chiquiticus gen. nov.

Taxon classificationAnimaliaColeopteraStaphylinidae

2254B309-2EC1-55C2-91F2-154CDD92BBD3

https://zoobank.org/4BCF12DF-8231-4269-B13B-917E194D43B9

Figs 1 , 2 , 3 , 4A–C

##### Type species.

*Heterothopspusio* J.L. LeConte, 1863, here designated.

##### Included species.

*Chiquiticusarizonicus* (Smetana, 1971), comb. nov. ex. *Heterothops* [holotype and 2 paratypes from CNC examined]; *Chiquiticuscampbelli* (Smetana, 1971), comb. nov. ex. *Heterothops* [holotype, and 7 paratypes from CNC examined]; †*Chiquiticuscornelli* (Chatzimanolis & Engel, 2013), comb. nov. ex. *Heterothops* [photos of the holotype and one paratype from AMNH examined]; *Chiquiticusgemellus* (Smetana, 1971), comb. nov. ex. *Heterothops* [holotype from MCZ and 2 paratypes from CNC 2 examined]; †*Chiquiticusinfernalis* (Chatzimanolis & Engel, 2013), comb. nov. ex. *Heterothops* [moved to *Chiquiticus* based on the data in the publication]; *Chiquiticusoccidentis* (Casey, 1886), comb. nov. ex. *Heterothops* [holotype of *Heterothopsmediocris* Fall, 1907, from MCZ examined, *H.mediocris* is a junior synonym of *H.occidentis* according to Smetana (1971) and non-type material identified by A. Smetana in CNC]; *Chiquiticuspusio* (LeConte, J. L., 1863), comb. nov. ex. *Heterothops* [1 presumed syntype or at least historical specimen examined, (MCZ) conspecific with the type material according to Smetana (1971) and non-type material identified by A. Smetana in CNC]; *Chiquiticusrambouseki* (Blackwelder, 1943), comb. nov. ex. *Heterothops* [1 syntype from NHM examined].

##### Diagnosis.

Small Staphylininae mainly around 2.5–3.5 mm long (Fig. 1A, B) with subconical head with ventral basal ridge but without postgenal ridge (Fig. 1C); without supra-antennal punctures (Fig. 4A); pronotum transverse (PW/PL ≥ 1.1), with its maximum width in posterior half, with paired punctures on dorsal series widely separated from each other (Fig. 4B); mesanapleural sutures transverse not reaching or fusing with external part of prepectus (Fig. 1F); males with black combs on first mesotarsomeres, but without black combs on mesotrochanters; tergites VII and VIII without broader, foliose setae in addition to usual acuminate, simple setae; tergite X not fused to lateral tergal sclerites in males.

##### Description.

Body small (TL = 2.3–3.9 mm). Head (Fig. 1C): frontoclypeal (epistomal) “suture” complete; head about as wide as long (HW/HL > 0.9 but < 1.1); neck from moderately (NW/HW > 0.75 but < 0.90) to distinctly wide (NW/HW ≥ 0.90); dorsal macrosetae: anterolateral clypeal punctures present; frontoclypeal punctures present; supra-antennal punctures absent; parocular punctures present as one on each side or absent; basal punctures present, single; posterior frontal punctures located posterior to temporal punctures; gular sutures separated, gula with distinct transverse basal impression; ventral basal ridge (VBR) present, postgenal ridge absent; postmandibular ridge (PMR) extended diagonally towards gula; infraorbital ridge extends to PMR; nuchal ridge absent or rudimentary, at most present as a linear impression but not a ridge; eye from medium large (EYL/HL ratio > 1/2 but < 3/4) to very small (EYL/HL ratio less than 3/10); antennomere 1 distinctly shorter than antennomeres 3 and 2 combined; male and female antennomere 11 at least two times longer than antennomere 10; left mandible with proximal, not bifid tooth; maxillary palps: palpomere 4 (apical) subulate, distinctly shorter than palpomere 2, palpomere 3 markedly dilated compared to palpomere 4; labial palps: palpomere 3 (apical) more or less cylindrical, distinctly narrower and longer than palpomere 2, parallel-sided and needle-shaped, palpomere 2 markedly dilated at apex.

Thorax (Fig. 1E, F): prothorax with slightly transverse pronotum (PW/PL ≥ 1.1) with one pair of setiferous punctures in dorsal series widely separated from each other, the distance between them being about equal to the distance between them and the lateral margin; sublateral setiferous punctures present or absent; sternacostal (transverse carina) ridge medially not protruding; antesternal membrane with distinct semisclerotized plate (Fig. 1E); pronotum and prosternum not fused in procoxal cavity, pronotosternal suture complete in cavity; pronotal hypomeron not setose, without postcoxal hypomeral process; upper probasisternum with or without pair of macrosetae. Mesothorax: mesoscutellum without posterior scutellar carina, without sub-basal ridge; elytron without humeral spines or spine-like setae, with evenly setiferous punctation on disc and epipleuron; mesanapleural sutures (Fig. 1F) transverse not reaching or fusing with external part of prepectus, medially fused or nearly touching each other; apex of mesobasisternal intercoxal process varies from sharply pointed to obtuse angle, without V-shaped projection medially; mesocoxal cavities contiguous; pericoxal ridge absent. Metathorax with wings present, with veins CuA and MP4 fused in one vein; metakatepisternal processes divided. Legs: protarsomeres expanded in both sexes; apical tarsomere of all legs with one empodial seta distinctly shorter than tarsal claws; protibial row of laterodorsal spines always present in females but maybe absent in males in some species; ventral tibial spur resting at base of apical excavation connected to tibial margin by thin membranous region; first segment of mesotarsi in males with black comb lateroventrally, without pale adhesive setae; mesotrochanters in both sexes without black comb.

Abdomen: tergites III–V with only anterior transverse basal carina; tergites III–VI with a large posteromarginal macroseta on each side (Fig. 1D); tergites VII and VIII without broader, foliose setae in addition to the usual acuminate, simple setae; anterior transverse basal carina of tergite VII not continuing to paratergites; lateral tergal sclerites IX cylindrical; male sternite VIII with medial apical emargination; male sternum IX symmetrical. Aedeagus with rounded apex.

##### Distribution.

Nearctic region, Central America, the Caribbean. Introduced into Central Europe: *C.pusio* in Germany (Schülke and Renner 2020) and another species related to *C.pusio* collected in Austria with a car net.

##### Ecology.

The North American species occur in various types of debris like leaf litter and similar substrates, with some species also occurring facultatively in mammal burrows or nests (*Neotoma* Say & Ord, 1825), especially in drier regions (Smetana 1971; Adam Brunke pers. comm.). In Europe, where they are introduced, they have been collected using car nets.

##### Comparison.

*Chiquiticus* gen. nov. can be distinguished from all other genera of Staphylininae in broad sense (Newton 2022) by the combination of characters mentioned in the diagnosis. From species close to the type of *Heterothops*, it is distinguished by the paired setiferous macropunctures of the dorsal series being distinctly separated from each other, by a black comb on first mesotarsomere of males, and by presence of only one posteromarginal macrosetae on each side of tergites III–VI. In species close to the type of *Heterothops*, the paired setiferous macropunctures of the dorsal series are close together, the distance between them being distinctly less than the distance between dorsal row and respective lateral margin of pronotum. Also, in the true *Heterothops*, males do not have a black comb on the first mesotarsomere and their pattern of posteromarginal large macrosetae on tergites III–VI is 1, 1, 2 and 2 per side. For more details on the differentiation between *Chiquiticus* gen. nov. and *Heterothops* species from Central Europe, see Schülke and Renner (2020). Presence of a frontoclypeal “suture” is a rare trait in Staphylininae, which in *Chiquiticus* gen. nov. is always visible. In Staphylininae this character is found in few basal lineages, like *Arrowinus* Bernhauer, 1935, several genera of Amblyopinina including *Loncovilius* Germain, 1903 (Reyes-Hernández et al. 2024), and in the relatives to *Chiquiticus*, *Amazonothops* (Jenkins Shaw et al. 2020), and *Ctenandropus*. Additionally, *Chiquiticus* gen. nov. displays an antesternal plate, currently known as a unique character of groups such as *Arrowinus* and several tribes united in Xatholininae sensu Żyła and Solodovnikov (2020). However, this plate sometimes is also found in Amblyopinina among Staphylinini, for example in species that are close to the types of the currently non-monophyletic genera *Heterothops* and *Cheilocolpus* (Reyes-Hernández et al. 2024). Furthermore, *Chiquiticus* exhibits unique characters shared only with the related genera *Ctenandropus* and *Amazonothops*, such as the presence of transverse mesanapleural sutures distinctly separated from the external portion of the prepectus (Fig. 1F and fig. 3E in Jenkins Shaw et al. (2020) misinterpreted as “transvere ridge (TR)”) and distinctly elongated protergal glands (fig. 3F in Jenkins Shaw et al. (2020)). *Chiquiticus* is distinguished from *Ctenandropus* by the subconical head with ventral basal ridge; the transverse pronotum (PW/PL ≥ 1.1) with its maximum width in the posterior half; usually by presence of intercoxal carina separating the upper probasisternum and lower probasisternum; and by tergites III–V lacking a posterior transverse carina. In *Ctenandropus*, the head is subquadrate, without postgenal ridge or ventral basal ridge; the pronotum is as long as wide (PW/PL > 0.9 but < 1.1), with its maximum width in the anterior half; without intercoxal carina separating the upper probasisternum and lower probasisternum; and tergites III–V have a posterior transverse carina. *Chiquiticus* is distinguished from *Amazonothops* by the absence of a black comb on the mesotrochanters of males and foliaceous setae on the tergites in both sexes, by distinctly short empodial setae, by the tergite X not fused to internal face of lateral sclerites and by paramere with rounded apex and short lateroapical setae.

##### Etymology.

The name is derived from the Latinization of the word “Chiquitico”, which is a term used in some Hispanophone countries to refer to very small things. The gender is masculine.

##### New combinations notes.

The extinct species †*C.infernalis* from Dominican Amber is here restudied based on the original descriptions only. Many details important for the taxonomic placement of this species (e.g. pronotum and mesanapleural sutures) are not visible in the photos of this fossil there and probably they would be available only with the mCT examination. However, such visible features as habitus including small body size, as well as antennal bases located close to each other, very elongated last antennomere that is almost as long as the two preceding ones, oval and setose preapical maxillary palpomere that is clearly wider than the apical one, and wide neck allowed us to conclude that †*C.infernalis* is an extinct member of the new genus *Chiquiticus*. For †*C.cornelli*, in addition to the original description, we were able to examine better photos of the holotype and paratype kindly made available at our request (Fig. 3). In these images, in addition to the characters previously described for †*C.infernalis*, the connection of the anterior tentorial arms (Fig. 3B) and the widely separated paired punctures in the dorsal series (Fig. 3C) are visible. It should be noted that both fossil species are known only from females. Also, two specimens of the extant *C.rambouseki* (Blackwelder, 1943) that we examined were females and not in the best condition; however, the combination of their visible characters allows the species to be placed in *Chiquiticus*.

##### Biogeographic note.

As far as currently known, the Nearctic is the region with the greatest species diversity of *Chiquiticus*. However, the *Chiquiticus* fossils found in mid-Miocene Dominican amber (Chatzimanolis and Engel 2013) could suggest that Nearctic speciation took place after the genus dispersed there from the southern hemisphere where its closely related genera *Amazonothops* and *Ctenandropus* are also found (Reyes-Hernández et al. in prep.). Such dispersal may have occurred via the GAARlandia land bridge (Iturralde-Vinent 2006; Masonick et al. 2017; Iturralde-Vinent and MacPhee 2023).

#### ﻿Genus Nitidocolpus gen. nov.

Taxon classificationAnimaliaColeopteraStaphylinidae

3699DF70-13BF-5ECB-952C-7177CE9A72D0

https://zoobank.org/9BC238C3-B742-4E1E-8B59-48E8C83288DF

Figs 5 , 6A–C

##### Type species.

*Quediuscolumbinus* Bernhauer, 1917, here designated.

##### Included species.

*Nitidocolpusaurofasciatus* (Bernhauer, 1917), comb. nov. ex. *Quedius* [1 syntype from FMNH examined]; *Nitidocolpuschampioni* (Sharp, 1884), comb. nov. ex. *Quedius* [1 syntype from FMNH examined]; *Nitidocolpuscolumbinus* (Bernhauer, 1917), comb. nov. ex. *Quedius* [2 syntypes from FMNH examined]; *Nitidocolpusgermaini* (Bernhauer, 1917), comb. nov. ex. *Quedius* [1 syntype from FMNH examined]; *Nitidocolpusillatus* (Sharp, 1884), comb. nov. ex. *Quedius* [1 syntype from NHM examined]; *Nitidocolpuslaeticulus* (Sharp, 1884), comb. nov. ex. *Quedius* [1 syntype from NHM examined]; *Nitidocolpustriangulum* (Fauvel, 1891), comb. nov. ex. *Quedius* [1 syntype from IRSNB examined].

##### Diagnosis.

Supra-antennal punctures and paraocular punctures absent (Fig. 6A); pronotum without paired dorsal series of setiferous macropunctures (Fig. 6B); mesanapleural sutures transverse or nearly transverse but reaching the external part of prepectus (Fig. 5E); mesobasisternal intercoxal process with V-shaped projection medially (Fig. 5E); metatrochanters with apical spine (Fig. 5F).

##### Description.

Small to medium-sized Staphylininae (TL = 4.5–10 mm) (Fig. 5A–D). Head: Frontoclypeal (epistomal) suture absent or distinct only laterally and only in teneral (or chemically cleared) specimens; head slightly transverse (HW/HL ≥ 1.1) without distinct posterior angles; disc smooth; frons with microsculpture as transverse waves, without distinct concavity between antennal insertions; neck moderately wide (NW/HW > 0.75 but < 0.90); dorsal macrosetae (Fig. 6A): anterolateral clypeal punctures present; frontoclypeal punctures present; supra-antennal punctures absent; parocular punctures absent; basal punctures present, single; ventral macrosetae: postocular punctures present; infraorbital punctures absent; postmandibular punctures present; submentum with two pairs of macrosetae; mentum with seta alpha only, seta beta absent; gular sutures separated from each other, gula with distinct transverse basal impression; ventral basal ridge present; postgenal ridge present; postmandibular ridge short, extended parallel to margin of eye, without fusion with any other ridge and distinctly separated from eye margin; infraorbital ridge merged with postgenal ridge; nuchal ridge missing dorsally, present laterally, merged with infraorbital ridge; eyes of medium size (EYL/HL ratio more than 1/2 but less than 3/4); gena short (GL/ETL < 0.5); tomentose pubescence begins from fourth antennomere, its density on fourth antennomere as on following antennomeres; setation of antennomere 3 almost evenly distributed; antennomere 11 with subapical rounded field, without subapical lateral pits; mandibles with dorso-lateral seta, without dorso-lateral groove, external edge with curved base and apex, straight in middle length; right mandible with proximal tooth and bifid distal tooth, apically deflexed ventrad at an angle of >20°; left mandible rather straight; labrum with transparent wide apical membrane, anterior margin emarginate at middle; maxillary palps: palpomere 4 (apical) subconical, with evenly narrowed apex, longer than palpomere 2 and weakly dilated compared to palpomere 3; labial palps: palpomere 3 (apical) more or less fusiform or subconical, distinctly longer than, and about as wide as, palpomere 2.

Thorax: Prothorax with slightly transverse pronotum (PW/PL ≥ 1.1), without dorsal or sublateral series of setiferous punctures; prosternum without longitudinal keel; antesternal membrane without distinct semisclerotized patch or patches; probasisternum triangular, with narrowed lateral arms and disc protruding medially, with pair of macrosetae on the upper probasiternum; with rounded postcoxal hypomeral process, interrupted by inferior line. Mesothorax: mesoscutellum without posterior scutellar carina, without sub-basal ridge; elytra with humeral spines or spine-like setae, with even setiferous punctation on disc and epipleuron (sometimes with various setose color patterns); with setiferous punctures at apical margin of elytral suture (underside); mesanapleural sutures transverse or nearly transverse but reaching and fusing with external part of prepectus; mesobasisternum with intercoxal process narrowly pointed into sharp angle, without V-shaped projection medially; mesocoxal cavities contiguous; pericoxal ridge present and complete. Metathorax: wings present, with veins CuA and MP4 fused; metakatepisternal processes divided; metascutellar mid-longitudinal internal suture well developed (only visible in chemically cleared specimens). Legs: apical tarsomere of all legs without dorsal setae, with one empodial seta distinctly shorter than tarsal claws; protarsomeres 1–3 distinctly wider than long in both sexes, with pale adhesive setae ventrally; procoxa with internal ridge running parallel along external ridge; mesotarsi in both sexes without black comb, sometimes in males ventral side of first mesotarsomere with pale adhesive setae; mesotibiae straight; metatarsomere 1 shorter than metatarsomere 5; metatarsi shorter than metatibiae; metatrochanter apically rounded, with strong straight dorsoapical spine; metacoxae with four or fewer spines on ventral posterolateral lobe, spines on dorsomedial disc also present; basal part of metacoxae distinctly wider and more convex than apical part.

**Figure 7.**
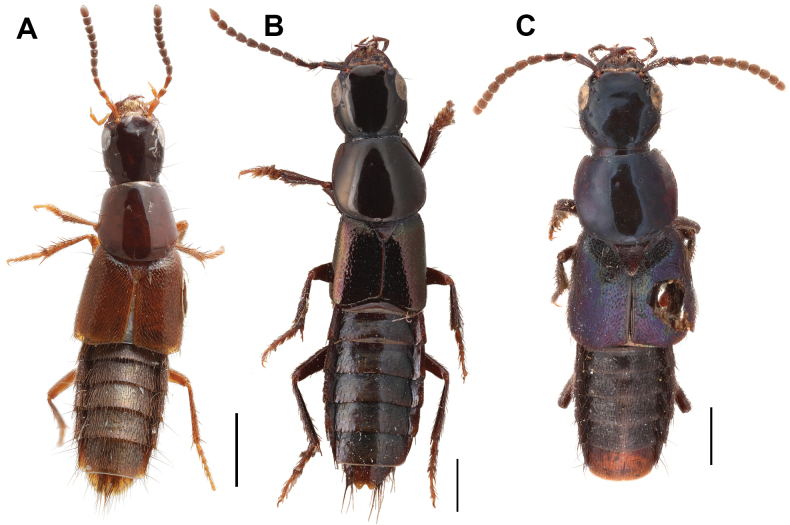
Some *Cheilocolpus* species **A***Ch.pyrostoma* (Solier, 1849) **B***Ch.speciosus* (Bernhauer, 1917) **C***Ch.viridulus* (Erichson, 1840).

Abdomen: protergal glands well manifested as rounded cuticular acetabula; tergites III–V in some species with posterior basal transverse carinae, in some species they are only with anterior carinae; on tergite VII anterior transverse basal carina not continuing to paratergites; tergites III–VI with different patterns of posteromarginal large macroseta (PMM) on each side, IV–VI with more than one PMM per side; punctation of tergites in form of fine to moderate impressions, some species with wide glabrous areas; lateral tergal sclerites IX short and slightly laterally flattened; male sternite VIII with medial apical emargination. Aedeagus with paramere fused to median lobe only at base and very closely appressed to median lobe along entire length; base of the paramere flat medially, not projecting upwards in the middle; paramere strongly produced over apex of median lobe. Ovipositor with each second gonocoxite with one medial macroseta, without spine-like setae on outer lateral margin.

##### Distribution.

Neotropical Realm: Mexican Transition Zone, Central America, and northern South America.

##### Ecology.

This genus has been collected from a variety of microhabitats, including mushrooms (e.g., *Pleurotus* spp.), under decomposing logs, beneath bark, and in leaf litter found in cloud, oak, and pine forests. They are also frequently captured using flight-intercept and Malaise traps. Additionally, some species are associated with the inflorescences of plants from the families Arecaceae, Betulaceae, and Heliconiaceae.

##### Comparison.

*Nitidocolpus* gen. nov. can be distinguished from all other genera of Staphylininae by the combination of characters mentioned in the diagnosis. *Nitidocolpus* is further distinguished from the superficially similar Quediina and Cytoquediina (Fig. 6) by the lack of supra-antennal punctures, the presence of a single carina on the mesoscutellar shield (posterior carina absent), by hind wings with fused CuA and MP4 veins, by a single empodial setae, and by the base of the paramere, which has no upward or forward projection in the middle. Compared to other Neotropical genera that resemble *Nitidocolpus*, such as *Cheilocolpus*, *Mimosticus* Sharp, 1884, and *Heterothops*, *Nitidocolpus* is distinguished by the absence of paraocular punctures on the head, the lack of setiferous punctures in or near the center of the pronotal disc, and the presence of postcoxal hypomeral processes. In some species of *Nitidocolpus*, such as *N.laeticulus* (Fig. 5B) and *N.aurofasciatus* (Fig. 5D), the basal punctures have shifted anteriorly and thus may be erroneously considered paraocular punctures. However, they can be easily distinguished as basal punctures, because they are the only punctures located near the posterior frontal punctures, aside from the temporal punctures. In other species of *Nitidocolpus*, such as *N.illatus* (Fig. 5A), the innermost macrosetae on the anterior margin maybe erroneously considered as a puncture of the dorsal row because it is located far from the anterior margin of pronotum.

##### Etymology.

The name is derived from the Latin words “nitidus” and “colpus”, which mean “shiny” and “hit”, respectively. The name refers to the great clarity with which the head and pronotum punctures are seen. The gender is masculine.

### ﻿New combinations in the genera *Cheilocolpus* Solier, 1849 and *Cyrtoquedius* Bernhauer, 1917

As shown above, describing the genus *Nitidocolpus* required careful examination of a number of the poorly known species of *Quedius*, some of which, as we should stress, do not belong to *Nitidocolpus*. They belong to neither *Quedius* nor Quediina. Our examination revealed that most of them belong to the amblyopinine genus *Cheilocolpus*, as it is defined in Coiffait and Sáiz (1966) and Sáiz (1971), with adjustments by Reyes-Hernández et al. (2024); i.e., they are close to the type species *Cheilocolpuspyrostoma* (Solier, 1849) (Fig. 7A). As a result, here we move these species as follows: *Cheilocolpusforsteri* (Scheerpeltz, 1960), comb. nov. ex. *Quedius* [although the type material was not examined, the detailed original description and distribution data provided by Scheerpeltz (1960) allow this species to be placed within *Cheilocolpus*; it has a combination of features typical for *Cheilocolpus* from the páramos: brachypterous habitus, entirely brownish-black body, rectangular head with transverse microsculpture and small convex eyes, paraocular punctures, and tergites III–V with posterior transverse basal carina]; *Cheilocolpusspeciosus* (Bernhauer, 1917), comb. nov. ex. *Quedius* [1 syntype from FMNH examined (Fig. 7B)]; *Cheilocolpusviridulus* (Erichson, 1840), comb. nov. ex. *Quedius* [2 syntypes from MFNB examined (Fig. 7C)].

Furthermore, we propose the following new combination for the species *Cyrtoquediusviridipennis* (Fauvel, 1891), comb. nov. ex. *Quedius*. Although the type material was not examined, the original description and distribution data provided by Fauvel (1891) support placing this species within *Cyrtoquedius*, in accordance with the diagnosis by Brunke et al. (2016). Fauvel noted that it is closely related to *Cy.labiatus* (Erichson, 1840) but differs in coloration. He also described the elytra as having three rows of larger punctures, with the remaining punctures faint and vaguely marked, which are typical characteristics of this genus. This contrasts with *Nitidocolpus* and *Cheilocolpus*, where the elytra are evenly setose rather than mostly glabrous except for rows of macrosetae.

## ﻿Discussion

The description of *Chiquiticus* gen. nov. and *Nitidocolpus* gen. nov. was necessitated due to the upcoming formal taxonomic embedding of the newly discovered lineages in Staphylininae in Reyes-Hernández et al. (in prep.). Also, it is a step towards badly needed taxonomic clean-up of the polyphyletic large wastebasket genera *Heterothops* (Amblyopinina) and *Quedius* (Quediina), especially for the New World fauna, because here we based our genus descriptions solely on the species earlier described in those genera. These are eight species of *Heterothops* (of which two are extinct) for *Chiquiticus* and eight species of *Quedius* for *Nitidocolpus*. Globally, many more non-related convergently similar species remain misplaced in both *Heterothops* and *Quedius*. In the American fauna, following the establishment of *Chiquiticus* and *Nitidocolpus*, the number of misclassified *Heterothops* species has significantly decreased. Moreover, in the course of this study we reclassified a number of the Neotropical *Quedius* species which superficially resemble *Nitidocolpus* but in fact, belong to the amblyopinine genus *Cheilocolpus* or the cyrtoquediine genus *Cyrtoquedius*. It should be noted that in both *Chiquiticus* and *Nitidocolpus* there are also several new species to be described; as well as in the genus *Cheilocolpus* and other genera of the former “southern quediines”. Due to the above-mentioned pragmatic taxonomic purposes of the current generic descriptions, here we considered solely the described species based on the type material or their original descriptions if they displayed enough data to adequately identify them as either new genus. Descriptions of all new species as well as the update of the taxonomy including lectotype designations, redescriptions, summary of the distribution, and bionomics information on the earlier described species in both new and other involved genera will be provided in future revisionary work.

## Supplementary Material

XML Treatment for Chiquiticus

XML Treatment for Nitidocolpus

## ﻿Additional information

### Author ORCIDs

José L. Reyes-Hernández https://orcid.org/0000-0002-4726-4439

Alexey Solodovnikov https://orcid.org/0000-0003-2031-849X

### Data availability

All of the data that support the findings of this study are available in the main text.
